# 非IgM型淋巴浆细胞淋巴瘤临床及生物学特征

**DOI:** 10.3760/cma.j.issn.0253-2727.2022.07.007

**Published:** 2022-07

**Authors:** 颖 于, 文婕 熊, 佳雯 陈, 阳 焦, 禹廷 阎, 齐 王, 德慧 邹, 薇 刘, 慧敏 刘, 瑞 吕, 录贵 邱, 树华 易

**Affiliations:** 中国医学科学院血液病医院（中国医学科学院血液学研究所），实验血液学国家重点实验室，国家血液系统疾病临床医学研究中心，细胞生态海河实验室，天津 300020 State Key Laboratory of Experimental Hematology, National Clinical Research Center for Blood Diseases, Haihe Laboratory of Cell Ecosystem, Institute of Hematology & Blood Diseases Hospital, Chinese Academy of Medical Sciences & Peking Union Medical College, Tianjin 300020, China

**Keywords:** 淋巴浆细胞淋巴瘤, 免疫球蛋白G, 免疫球蛋白A, 临床特征, 细胞遗传学, Lymphoplasmacytic lymphoma, Immunoglobulin G, Immunoglobulin A, Clinical characteristics, Cytogenetics

## Abstract

**目的:**

探索非IgM型淋巴浆细胞淋巴瘤（LPL）患者的临床及生物学特征。

**方法:**

回顾性收集中国医学科学院血液病医院1993年7月至2020年8月收治的340例LPL患者的临床资料，其中23例为非IgM型LPL组，317例为华氏巨球蛋白血症（WM）组。比较两组患者的临床及生物学特征。

**结果:**

23例非IgM型LPL患者中，2例分泌单克隆性IgA，14例分泌单克隆性IgG，7例不分泌单克隆性免疫球蛋白。非IgM型LPL和WM患者中位年龄均为62（35～81）岁。与WM组患者相比，非IgM型LPL组患者女性（56.5％对27.3％，*P*＝0.007）、脾大（60.1％对43.8％，*P*＝0.100）、结外侵犯（21.7％对12.3％，*P*＝0.672）比例更高。非IgM型LPL组18例患者进行了MYD88基因相关检测，阳性率55.6％。非IgM型LPL组17例患者接受了治疗，启动治疗的患者比例与WM组患者相当（94.4％对92.7％，*P*＝0.488）。非IgM型LPL组16例患者进行了疗效评价，一线治疗总体缓解率87.5％，中位随访时间33.9（3.5～125.1）个月，总体中位无进展生存（PFS）、总生存（OS）时间未达到，3年PFS率和OS率分别为71.4％和68.9％。WM组中位PFS、OS时间分别为66.2个月和78.1个月。两组PFS、OS的差异均无统计学意义（*P*值分别为0.340、0.544）。

**结论:**

非IgM型LPL与WM患者的临床及生物学特征相似，但非IgM型LPL组女性、结外受累比例更高。非IgM型LPL患者的生存及预后与WM患者相似。

## Abstract (en)

**Objective:**

The study aims to explore the clinical and biological characteristics of patients with non-IgM lymphoplasmacytic lymphoma（LPL）.

**Methods:**

The clinical data of 340 patients with LPL admitted to the Institute of Hematology & Blood Diseases Hospital, Chinese Academy of Medical Sciences & Peking Union Medical College were collected retrospectively, including 23 cases of the non-IgM LPL and 317 cases of the Waldenström's macroglobulinemia（WM）, from July 1993 to August 2020. The clinical and biological characteristics of the two groups were compared.

**Results:**

Among 23 patients with the non-IgM type LPL, two patients secreted monoclonal IgA, 14 patients secreted monoclonal IgG, and seven patients did not secrete monoclonal immunoglobulin. The median age of the non-IgM LPL and WM were both 62（35−81）years old. Compared with the WM group, the proportion of women（56.5％ *vs* 27.3％, *P*＝0.007）, the proportion of splenomegaly（60.1％ *vs* 43.8％, *P*＝0.100）, and the proportion of extranodal invasion（21.7％ *vs* 12.3％, *P*＝0.672）in non-IgM LPL group were higher. Eighteen patients were tested for MYD88 gene mutation, and the overall mutation rate of MYD88 was 55.6％. In the non-IgM LPL group, a total of 17 patients received treatment, which had a comparable proportion（94.4％ *vs* 92.7％, *P*＝0.488）to the WM group. Sixteen patients were evaluated for efficacy, and the overall remission rate of the first-line treatment was 87.5％. The median follow-up time was 33.9（3.5–125.1）months, and the median PFS and OS were both not reached. The 3-year PFS and OS rates were 71.4％ and 68.9％, respectively. In the WM group, the median PFS was 66.2 months and the median OS was 78.1 months. Compared with the WM group, in the non-IgM group no significant differences in PFS（*P*＝0.340）and OS（*P*＝0.544）were seen.

**Conclusion:**

The clinical and biological characteristics of the non-IgM LPL and WM patients were similar. However, the proportion of women and extranodal involvement were higher in the non-IgM LPL group. The survival and prognosis of the non-IgM LPL patients were similar to those of the WM patients.

淋巴浆细胞淋巴瘤（LPL）是一种罕见的惰性B细胞淋巴瘤，国外报道显示，其在非霍奇金淋巴瘤（NHL）中占1％～2％[1]。LPL通常由小B淋巴细胞、浆样淋巴细胞和浆细胞组成[1]，多数累及骨髓，也可累及淋巴结和脾脏。大部分LPL患者分泌单克隆性IgM成分，但仍有少部分患者分泌IgG、IgA或不分泌单克隆免疫球蛋白。LPL侵犯骨髓并分泌单克隆免疫球蛋白IgM时被称为华氏巨球蛋白血症（Waldenström's macroglobulinemia, WM），其他为非IgM型LPL。我们对LPL的研究主要集中在WM上，而对非IgM型LPL知之甚少。国内对非IgM型LPL的研究主要是个案报道，国外也无大系列报道。本研究对中国医学科学院血液病医院23例非IgM型LPL患者的临床、生物学特征进行了回顾性分析，并与WM患者进行比较，以探索两者的异同，提高大家对非IgM型LPL的认识。

## 病例与方法

1. 病例：回顾性分析中国医学科学院血液病医院淋巴瘤诊疗中心1993年7月至2020年8月收治的340例LPL患者，其中23例为非IgM型LPL。非IgM型LPL的诊断标准依据最新修订的WHO淋巴与造血组织肿瘤诊断标准[2]。WM的诊断依据第二届国际WM工作组标准[3]。23例非IgM型LPL患者均行骨髓病理学检查确诊，1例患者同时行骨髓及扁桃体病理学检查确诊，所有患者均进行单克隆免疫球蛋白定性及定量检测。收集的临床资料包括：患者的性别，初诊年龄，首发症状，血常规、生化常规、免疫功能、乙型肝炎病毒抗原及抗体等实验室检查结果，骨髓细胞形态学、骨髓病理、流式细胞术、FISH、染色体、二代基因测序、MYD88突变等检查结果，彩超、CT等影像学检查结果，以及治疗方案、疗效、预后、随访时间等。

2. 疗效评价：非IgM型LPL及WM疗效评价参照第六届LPL/WM国际研讨会制定的标准进行评估（表1）。对于不分泌型LPL暂无统一的评价标准，主要参照非分泌型多发性骨髓瘤，结合骨髓中瘤细胞数量、免疫球蛋白定量及影像学检查进行综合评估。

**表1:** 华氏巨球蛋白血症（WM）的疗效评价标准

疗效	评价标准
完全缓解（CR）	两次免疫固定电泳阴性（间隔6周以上），IgM正常；无骨髓受累；原有髓外病灶消失，如淋巴结或脾脏肿大；无WM相关临床症状及体征
非常好的部分缓解（VGPR）	M蛋白下降≥90％；原有髓外病灶消失，如淋巴结或脾脏肿大；无疾病活动
部分缓解（PR）	M蛋白下降50％～90％；原有髓外病灶缩小≥50％，如淋巴结或脾脏肿大；无疾病活动
微小反应（MR）	M蛋白下降≥25％但<50％；无疾病活动
疾病稳定（SD）	M蛋白增加或减少<25％；淋巴结肿大、脏器肿大、WM相关症状无进展
疾病进展（PD）	经2次鉴定，M蛋白增加≥25％；或由本病所致临床表现或体征加重

3. 随访：采用电话对患者进行随访。随访截至2020年9月，19例患者完成随访，4例失访，中位随访时间33.9（3.5～125.1）个月。无进展生存（PFS）期定义为从诊断到疾病进展、死亡或末次随访的时间。总生存（OS）期定义为开始治疗到死亡或末次随访的时间。总有效率（ORR）定义为获得完全缓解（CR）、非常好的部分缓解（VGPR）、部分缓解（PR）、微小反应（MR）患者所占比例。

4. 统计学处理：采用SPSS 26.0软件进行统计学分析，计数资料用例数（百分比）表示，计量资料用中位数（范围）表示。计数资料的组间比较采用*χ*^2^检验和Fisher确切概率法，计量资料的比较采用*t*检验。生存分析采用Kaplan-Meier法，并用双侧对数秩检验进行比较，*P*<0.05定义为差异具有统计学意义。

## 结果

1. 临床基线资料：研究纳入340例LPL患者，其中317例（93.2％）分泌单克隆性IgM且均伴有骨髓侵犯，诊断为WM，其中6例（1.8％）同时分泌单克隆性IgM和IgG[4]，无仅分泌IgM不侵犯骨髓的患者。非IgM型LPL患者23例（6.8％），包括分泌单克隆性IgA 2例（0.6％，均为κ轻链），分泌单克隆性IgG 14例（4.1％，9例为κ轻链，5例为λ轻链），不分泌单克隆性免疫球蛋白7例。

2例IgA型患者IgA定量分别为35.4 g/L和72.6 g/L，IgM均明显减低（0.14 g/L和0.33 g/L）。14例IgG型患者中位IgG水平31.8（19.9～92.5）g/L，中位M蛋白水平21.0（10.6～65.0）g/L，除2例患者IgM轻度升高（3.73 g/L、4.33 g/L）外，IgM均减低；7例不分泌型患者中，4例IgM定量4.2～8.2 g/L，高于正常值上限，其余3例患者IgG、IgA、IgM均在正常范围，所有不分泌型患者均进行了2次血清蛋白电泳及免疫固定电泳验证。

23例非IgM型LPL患者中位年龄62（35～81）岁，其中男10例，女13例。淋巴结肿大或脾脏肿大患者分别为13例（56.5％）和14例（60.1％）。5例（21.7％）患者有结外侵犯：肾脏3例（13.0％），骨骼1例（8.7％），心脏1例（4.3％）。以贫血、乏力起病15例（65.2％），以双下肢、眼睑水肿起病3例（13.0％），以泡沫尿起病3例（13.0％），以双下肢麻木起病1例（4.3％），以血小板减少起病1例（4.3％）。8例患者出现B症状，各有1例患者出现周围神经炎及神经性耳聋。

2. 实验室检查：非IgM型LPL患者中，18例患者贫血，中位HGB水平为80.0（48.0～155.0）g/L。11例患者出现白细胞异常，其中9例减低，2例升高，中位WBC 4.7（1.4～28.4）×10^9^/L。9例患者出现PLT异常，其中8例减低，1例升高，中位PLT水平136（14～376）×10^9^/L。

5例患者出现肌酐升高，最高值为159 µmol/L，均为慢性肾脏病（CKD）3期。2例（8.7％）患者出现淀粉样变，分别累及肾脏及心脏。2例患者同时合并乙型肝炎（8.7％），表现为乙型肝炎表面抗原、e抗体、核心抗体阳性。1例患者同时合并继发性骨髓纤维化及冷凝集素升高。

所有患者均有骨髓受累，骨髓细胞形态学中位淋巴细胞/浆样淋巴细胞比例为57％（14％～94％）。18例患者进行了流式细胞术检查，中位异常细胞比例为24.46％（0.04％～98.30％）。15例（83.3％）患者呈CD5^−^CD10^−^（2例患者CD5弱表达，1例患者CD10部分表达）；11例患者呈CD38阳性（61.1％）；8例患者进行了CD138检测，均为阳性。

3. 遗传学检查：共有21例患者进行了FISH检测，5例（23.8％）患者可见FISH异常。21例患者进行了17p13（TP53）缺失检测，均未见异常。14例患者进行了11q22（ATM）缺失检测，均未见异常。18例患者进行了14q32（IGH）易位检测，3例阳性（16.7％），CCND1/IGH和BCL2/IGH易位均阴性。14例患者进行了13q14（RB-1）缺失检测，1例阳性（7.1％）。4例患者进行了CEP12检测，2例（50％）阳性。1例患者进行del（6q）（MYB）检测且为阳性。

18例患者应用等位基因特异性寡核苷酸聚合酶链反应（ASO-PCR）检测MYD88 L265P突变，7例阳性（38.9％）。共8例患者进行了二代基因测序，7例（87.5％）MYD88突变阳性，ASO-PCR结合二代测序共检出10例（55.6％）阳性。8例进行二代测序的患者中2例（25％）CXCR4突变阳性，其他突变基因包括TNFAIP3、KRAS、ACD、DNMT3B、FGFR3、ATG2B、EP300、ASXL1、TP53、FAT1、NOTCH1、PLCG2、DNMT3A。3例ASO-PCR阴性患者同时进行了二代测序检测，均发现MYD88 L265P突变。

4. 治疗及疗效：共17例患者接受治疗，1例未接受治疗，5例未获得治疗相关信息。在接受治疗的17例患者中，15例采用以新药（利妥昔单抗、硼替佐米、伊布替尼）为基础的方案，2例采用传统治疗方案［CHOP（环磷酰胺+长春新碱+阿霉素+泼尼松）样方案、沙利度胺］；6例患者进行了维持治疗。新药治疗组1例患者应用伊布替尼。各有7例采用以利妥昔单抗为基础和硼替佐米为基础的方案。16例患者进行了疗效评价，3例达到CR，2例达到VGPR，6例PR，3例MR，2例疾病稳定，一线治疗ORR为87.5％（表2）。

**表2:** 17例接受治疗的非IgM型淋巴浆细胞淋巴瘤患者治疗方案及疗效

例号	治疗方案	维持方案	最佳疗效	是否复发进展	PFS期（月）	OS期（月）
1	RFCD×4，COP×2	R×4	CR	否	125.1	125.1
2	FC×1，CHOP×5	否	PR	否	102.4	102.4
3	RCOP×8	否	CR	失访	−	−
4	BCD×7	沙利度胺	PR	是	30.4	33.5
5	BCD×4，BD×2	R×5	CR	否	60.4	60.4
6	TCD×8，VCR×3	沙利度胺	PR	否	56.7	56.7
7	BCD×4，RCD×6	否	PR	是	14.0	29.6
8	BCD	−	−	是	21.0	24.0
9	R-CDOP×5	否	VGPR	否	20.1	20.1
10	RCD×1，R×13	−	−	否	36.6	36.6
11	RCD×8	TP	SD	否	36.2	36.2
12	RCOP×5	TP	VGPR	否	34.9	34.9
13	BCD×5	沙利度胺	MR	否	33.9	33.9
14	R-CHOP×1，CD×2	否	MR	否	1.0	6.2
15	VRD×6	否	−	否	15.9	15.9
16	BCD	−	−	否	12.9	12.9
17	RCD×2，伊布替尼×7	否	否	否	9.1	9.1

注：PFS：无进展生存；OS：总生存；RFCD：利妥昔单抗+氟达拉滨+环磷酰胺+地塞米松；RCOP：利妥昔单抗+环磷酰胺+长春新碱+泼尼松；FC：氟达拉滨+环磷酰胺；CHOP：环磷酰胺+多柔比星+长春新碱+泼尼松；BCD：硼替佐米+环磷酰胺+地塞米松；BD：硼替佐米+地塞米松；TCD：沙利度胺+环磷酰胺+地塞米松；VCR：长春新碱；RCD：利妥昔单抗+环磷酰胺+地塞米松；R：利妥昔单抗；R-CDOP：利妥昔单抗+环磷酰胺+脂质体阿霉素+长春新碱+泼尼松；R-CHOP：利妥昔单抗+环磷酰胺+多柔比星+长春新碱+泼尼松；VRD：硼替佐米+利妥昔单抗+地塞米松；CR：完全缓解；PR：部分缓解；VGPR：非常好的部分缓解；SD：疾病稳定；MR：微小反应；−：未获得

5. 生存与预后：可获得随访结果的19例患者中，14例（73.7％）存活，5例（26.3％）死亡患者均死于疾病复发进展。总体中位PFS、OS时间未达到，3年PFS率、OS率分别为71.4％、68.9％。非IgM型LPL组患者的PFS、OS与WM组相比差异均无统计学意义（*P*值分别为0.340、0.544）（图1）。5例死亡患者情况如下：例4 IgG 27.8 g/L，血M蛋白19.2 g/L，FISH可见IGH基因扩增，予BCD方案7个疗程达PR，后予沙利度胺维持，OS时间33.5个月；例7染色体提示t（1;10），二代测序显示TNFAIP3、KRAS、ACD、DNMT3B基因突变，予BCD方案4个疗程后达到PR，后疾病进展予RCD方案6个疗程，达到PR，疾病再次进展，第一次疾病进展时间为14个月，第二次疾病进展时间为10个月，OS时间29.6个月；例8出现心肌淀粉样变，FISH提示RB基因缺失，OS时间24.0个月；例14继发骨髓纤维化，存在巨脾，二代测序提示MYD88、FAT1、NOTCH1基因突变，予R-CHOP及RCD方案达到MR，后因严重肺部感染死于呼吸衰竭，OS时间6.2个月；例15 FISH提示+12及IGH基因重排，IgG 80.3 g/L，血M蛋白41.7 g/L，OS时间15.9个月（表2）。

**图1:**
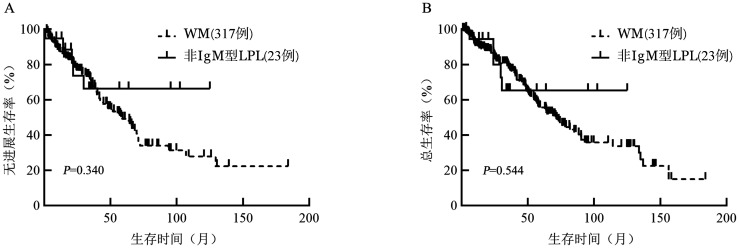
非IgM型淋巴浆细胞淋巴瘤（LPL）组与华氏巨球蛋白血症（WM）组患者的无进展生存（A）和总生存（B）曲线

23例非IgM型LPL中16例为分泌型，7例为不分泌型。与不分泌型患者相比，分泌型患者中位年龄大（64岁对55岁），淋巴结肿大比例（43.8％对28.6％）、脾大比例（62.5对57.1％）、MYD88阳性率（77.8％ 对20.0％）更高，而结外受累比例（12.5％对42.9％）更低，且中位HGB（79 g/L对85.0 g/L）、中位β_2_-微球蛋白（3.5 g/L对4.08 g/L）及中位白蛋白水平（32.4 g/L对39.4 g/L）更低。因样本量小，暂未行进一步统计学分析。

进一步将23例非IgM型LPL患者与317例WM患者的临床及生物学特征进行比较。两组患者临床及生物学特征相似，与WM组患者相比，非IgM型LPL组女性比例较高（56.5％对27.3％，*P*＝0.007）；同时脾大（60.1％对43.8％，*P*＝0.133）、结外受累（21.7％对12.3％，*P*＝0.327）、乳酸脱氢酶异常（21.7％对15.8％，*P*＝0.053）比例高，但差异均无统计学意义。WM组球蛋白水平较非IgM型LPL组更高（50.4 g/L对23.9 g/L，*P*＝0.306），差异无统计学意义。非IgM型LPL组和WM组分别有17例及254例患者接受治疗，启动治疗患者比例基本相当［94.4％（17/18）对92.7％（254/274），*P*＝0.781］，但非IgM型LPL组更多患者采用了以利妥昔单抗、硼替佐米、伊布替尼为基础的新药治疗方案（*P*＝0.044）（表3）。

**表3:** 非IgM型LPL组与WM组患者的临床特征及预后比较

特征	非IgM型LPL（23例）	WM（317例）	*χ*^2^值/*t*值^a^	*P*值
年龄［岁，*M*（范围）］	62（35～81）	62（28～87）	−1.503	0.134
男性［例（％）］	10（43.5）	229（72.2）	7.174	0.007
脾大［例（％）］	14（60.1）	139（43.8）	2.510	0.133
淋巴结肿大［例（％）］	13（56.5）	180（56.8）	0.000	1.000
结外受累［例（％）］	5（21.7）	39（12.3）	0.961	0.327
骨髓受累［例（％）］	23（100）	308（97.2）	0.596	0.884
HGB［g/L，*M*（范围）］	80.0（48～155）	85（24～187）	0.205	0.838
β_2_-微球蛋白［mg/L，*M*（范围）］	3.94（0.20～8.89）	3.87（0.23～25.60）	−0.890	0.929
血清白蛋白［g/L，*M*（范围）］	34.8（15.8～49.8）	33.6（11.9～48.8）	0.781	0.436
乳酸脱氢酶异常［例（％）］	5（21.7）	41（15.8）	1.321	0.053
血清球蛋白［g/L，*M*（范围）］	23.9（16.7～105.8）	50.4（2.4～131.0）	−1.026	0.306
轻链类型			1.295	0.332
κ型［例（％）］	11（68.8）	255（80.4）		
λ型［例（％）］	5（31.3）	62（19.6）		
MYD88 L265P突变阳性［例（％）］	10（55.6）	89（53.6）	0.025	0.675
随访时间［月，*M*（范围）］	33.9（3.5～125.1）	41.7（1.0～184.1）	0.269	0.747
接受治疗［例（％）］	17（94.4）	254（92.7）	0.105	0.781
新药组	15（88.2）	144（56.7）	5.302	0.044
以硼替佐米/伊沙佐米为基础	7（41.2）	48（33.3）		
以利妥昔单抗为基础	7（41.2）	89（61.8）		
伊布替尼/泽布替尼	1（5.9）	7（4.9）		
非新药组	2（11.8）	110（43.3）		

注：LPL：淋巴浆细胞淋巴瘤；WM：华氏巨球蛋白血症；^a^计数资料统计量为*χ*^2^值，计量资料统计量为*t*值

## 讨论

95％ LPL患者可分泌单克隆IgM并累及骨髓，这些病例被定义为WM[5]，大部分研究主要集中于WM，对非IgM型LPL罕有报道。本研究回顾性分析我中心收治的LPL患者，23例为非IgM型，占6.8％，与既往报道基本相符，也是目前已知的国内最大系列报道。

WM是一种罕见惰性淋巴瘤，白色人种年发病率（4.1/1 000 000）高于其他人种（1.8/1 000 000）[6]。2021年一项多中心WM回顾性研究显示，WM患者诊断时中位年龄63岁，男性多于女性（2.7∶1），中位随访32个月，预计3年OS率为82.7％[7]。2014年本中心回顾性分析了90例WM患者，30％患者出现淋巴结肿大，28.9％患者出现脾肿大，中位随访41个月，预计中位OS时间135个月，预计5年OS 率61.8％[4]。在本研究中，非IgM型LPL组患者中位年龄（62岁）与文献报道相似，脾大比例（60.1％）高于文献报道。

2019年，意大利一项研究纳入45例非IgM型LPL患者，与WM患者相比，非IgM型LPL患者女性比例更高，且更易出现淋巴结肿大、脾大及结外侵犯[8]，PFS及OS未见差异。美国MD安德森癌症中心的一项研究纳入17例非IgM型（9例IgG型，8例IgA型）LPL患者及312例WM患者，与WM组相比，非IgM型LPL组1年死亡率更高（23.5％对2.2％，*P*<0.01），OS率更低（76.5％对97.8％，*P*＝0.024），PFS未见差异[9]。韩国的一项研究纳入8例非IgM型LPL及22例WM患者，显示非IgM型LPL更易出现结外侵犯，OS时间更短（10个月对未达到，*P*＝0.05）[10]。

本研究显示，非IgM型LPL患者中女性比例高，更易出现脾大及结外受累，与部分文献报道一致。患者更易出现脾大提示诊断时需结合MYD88 L265P突变情况等与脾边缘区淋巴瘤进行鉴别诊断。总体上，非IgM型LPL患者的临床特征WM相似。非IgM型LPL与WM均为惰性淋巴瘤，生存时间长，预后较好。本研究中非IgM型LPL组3年OS率为68.9％，较WM患者更低，考虑与该组患者髓外受累比例更高有关，美国MD安德森癌症中心的研究也有同样结论[9]。

细胞遗传学结果显示，5例患者FISH检测异常。根据既往报道，WM中最常见的细胞遗传异常是6号染色体缺失，进一步FISH分析表明6q23-24.3为最小的缺失片段，其他常见的遗传学异常包括：+18、13q14（RB-1）缺失、+4、17p13（TP53）缺失、11q22（ATM）缺失、+12及14q32（IGH）易位[11]。然而，关于非IgM型LPL患者细胞遗传学的报道有限。曾有学者报道2例非分泌型LPL患者存在t（9;14），认为其可能是该类型患者的生物学标志[12]，而2004年有研究报道，t（9;14）在LPL患者中并不常见，也并非LPL的特征[13]。本研究同样检测出6q缺失、+12、17p13缺失及14q32易位。

MYD88 L265P突变在WM中常见，发生率达90％以上[14]–[16]，但并非WM特有，在其他淋巴瘤中也有MYD88突变的报道[17]–[19]。在WM患者中应用二代测序技术还可检测到CXCR4（30％～40％）、ARID1A（17％）和CD79B（8％～15％）等基因突变及6q染色体相关片段拷贝数改变[20]。2016年美国梅奥诊所的一项研究分析了非IgM型LPL的MYD88突变与临床及病理特征之间的关系，发现非IgM型LPL的MYD88突变率（约40％）明显低于WM，并未发现突变状态与临床及病理特征之间有明确相关性[21]。上文提到的意大利研究中，非IgM型LPL患者MYD88 L265P突变率显著低于WM患者（42％对91％）[8]。我中心23例非IgM型LPL患者中，MYD88 L285P突变阳性率为58.7％，略高于文献报道。MYD88突变率既往多采用ASO-PCR方法检测，该方法敏感性较低。本研究采用ASO-PCR及二代测序法检测MYD88突变，检出率分别为38.9％、87.5％，3例患者ASO-PCR法检测MYD88阴性而二代测序法检测阳性。可见二代测序可提高MYD88 L265P检出率，对肿瘤负荷低的患者更有优势。同时，二代测序可检出WM患者中常见的CXCR4、TP53突变。染色体核型、FISH及二代测序结果表明，非IgM型LPL患者可检出WM患者常见的遗传学异常，提示非IgM型LPL与WM存在共同的细胞遗传学异常。

综上所述，非IgM型LPL的临床及细胞遗传学特征与WM基本相似但仍有部分差异，采用WM的治疗方案，其生存及预后与WM患者相似。未来我们需要从基因组及转录谱等方面进一步研究，并具体分析非IgM型LPL与WM在治疗方案及疗效上的差异。
